# Going to the source: pancreatic lymph nodes maintain stem-like CD8^+^ T cells in human type 1 diabetes

**DOI:** 10.1172/JCI209019

**Published:** 2026-09-15

**Authors:** Fatoumata Samassa, Sylvaine You, Roberto Mallone

**Affiliations:** 1 Université Paris Cité, Institut Cochin, CNRS, INSERM, Paris, France.; 2 Indiana Biosciences Research Institute, Indianapolis, Indiana, USA.; 3 Assistance Publique Hôpitaux de Paris, Service de Diabétologie et Immunologie Clinique, Cochin Hospital, Paris, France.

## Abstract

Where does autoimmune persistence reside in type 1 diabetes (T1D)? Detailed investigation of the pancreas and pancreatic lymph nodes (PLNs) may provide more definitive answers, yet most studies in these target tissues have been confined to mouse models. In nonobese diabetic (NOD) mice, disease development is prevented by PLN ablation, and these LNs harbor a stem-like population of autoreactive CD8^+^ T cells. In this issue, Peters et al. identified stem-like CD8^+^ T cell populations enriched in PLNs from humans with T1D and provide evidence for a developmental continuum linking lymphoid and pancreatic immune compartments. Their findings extend concepts previously established in NOD mice and in cancer immunology to humans with T1D, wherein self-renewing T cell populations sustain long-term immune responses. While the precise relationship between these stem-like cells and pathogenic autoreactive clones remains unresolved, this work positions the PLN as a potential reservoir of autoimmune persistence in T1D and a target for immune intervention.

## Pancreatic lymph nodes: a reservoir for autoreactive T cells?

Type 1 diabetes (T1D) is a disease of pancreatic islet β cell destruction that is mediated by CD8^+^ T cells, yet most mechanistic studies rely on measurements of autoimmune T cells in peripheral blood, well distant from the battlefield of the pancreas. Moreover, peripheral blood is characterized by a state of so-called “benign” islet autoimmunity, in which autoimmune T cells circulate at similar frequencies in all individuals, irrespective of T1D status, whereas the same T cells are enriched in the pancreas of patients with T1D (1). On the one hand, this phenomenon calls for a more comprehensive phenotypic analysis of autoimmune circulating T cells to identify disease-specific signatures and the molecular switch from benign to pathogenic autoimmunity. On the other hand, it invites us to look more closely at the target organ, the pancreas. Research networks spearheaded by the Network for Pancreatic Organ Donors with T1D (nPOD) have made studies of the T1D pancreas possible by distributing such specimens.

More recently, there has been a revival of interest in pancreatic lymph nodes (PLNs) as key anatomical sites of the autoimmune process. This interest is rooted in older observations that PLN ablation at an early age prevents diabetes almost completely in the nonobese diabetic (NOD) mouse model of T1D (2). The role of PLNs in T1D pathogenesis has classically been linked to the priming of autoreactive T cells following β cell antigen drainage from the pancreas. More recently, A. Schietinger’s group broadened this view by identifying a population of stem-like autoreactive CD8^+^ T cells residing in PLNs of NOD mice (3). These T cells are at an intermediate state of differentiation, between naive and effector, and continuously replenish the pool of T cells migrating to the pancreas, where they acquire a more differentiated effector state mediating β cell cytotoxicity. This concept that autoimmune maintenance may depend on a self-renewing cellular reservoir rather than on terminal effectors alone is borrowed from the cancer immunology field, where these stem-like CD8^+^ T cells drive immune responses against tumor cells and are the main target of checkpoint inhibitor therapies, e.g., programmed cell death 1–blocking (PD-1–blocking) Abs (4). Yet, the intriguing suggestion that the same paradigm may operate in reverse in T1D has thus far remained confined to mouse models.

## A stem-like CD8^+^ T cell population in T1D PLNs

In this issue of the *JCI*, Peters et al. (5) present a comprehensive multimodal analysis of human PLNs from organ donors with and without T1D to provide evidence that a role for stem-like CD8^+^ T cells in T1D pathogenesis may indeed extend to humans. The authors sequentially applied complementary omics strategies — mass cytometry, single-cell RNA and T cell receptor (TCR) sequencing, flow cytometry, and spatial proteomics — on PLN specimens. Compared with PLNs from non-T1D donors, PLNs from T1D donors were enriched in naive-like CD8^+^ T cell clusters that displayed features consistent with stemness, notably increased surface CXCR3 expression, a marker previously also found enriched in circulating CD8^+^ T cells (6) that may confer pancreas-homing properties via secretion of CXCL10 by inflamed β cells (7). These stem-like CD8^+^ T cells combined transcriptional hallmarks of stemness (i.e., BCL2, BCL3, BCL6), reduced terminal differentiation and exhaustion, and molecular footprints of prior activation (NKG2D, DAP10), yet surprisingly lacked evidence of TCR clonal expansion. When comparing paired PLN and pancreas specimens from a more limited set of T1D donors, few shared TCRs were found, in line with previous reports. Nevertheless, PLNs from this T1D donor subset were enriched in CD8^+^ T cells with naive-like and stemness-associated features, whereas the same T cells isolated in the pancreas displayed a more differentiated effector/exhaustion signature.

These observations support a model in which less differentiated T cells are maintained within lymphoid tissue and subsequently acquire enhanced effector characteristics and eventual exhaustion after entering the inflamed pancreas, consistent with observations made in NOD mice (3). Spatial analysis strengthened this interpretation: T cell factor 1(TCF1^+^) and/or thymocyte selection-associated high-mobility group box (TOX^+^) CD8^+^ T cells were identified in both PLNs and pancreas, with TCF1^+^TOX^+^ double-positive cells localizing preferentially near islets. This phenotype is particularly interesting, because TCF1 and TOX have emerged as reciprocal regulators of progenitor and exhausted states, respectively. Rather than representing mutually exclusive programs, stemness and exhaustion may thus coexist within transitional cell populations. Whether such T cells represent pathogenic intermediates, regulatory checkpoints, or both remains an open question.

## Therapeutic implications: targeting PLN stem-like CD8^+^ T cells

These findings fit well with the repeated observation that clinical benefit from immunotherapies, particularly the anti-CD3 mAb teplizumab, correlates with induction of an exhausted phenotype in circulating CD8^+^ T cells (8). However, this exhausted phenotype is lost 12–18 months after treatment (8), while the delay in disease progression can be longer, raising the question of whether teplizumab only targets differentiated effector CD8^+^ T cells in the blood and/or pancreas, or also the progenitor stem–like CD8^+^ T cell reservoir in PLNs. Supporting the possibility of a dual effect, Wu et al. (9) recently reported that anti-CD3 treatment of NOD mice cleared islets from activated, clonally expanded cytotoxic T cells. Islets were subsequently reinfiltrated by more quiescent, clonally diverse, noncytotoxic CD8^+^ T cells that expressed increased levels of exhaustion-associated TOX. However, they also expressed increased levels of stemness-associated TCF1, and TCF1 expression did not decline along differentiation, in contrast to that observed in untreated mice. These findings suggest a hybrid stem-like/exhausted phenotype similar to that reported by Peters et al. (5).

Using transcriptional cytokine-response enrichment analyses, Peters et al. went on to implicate IL-15 as a candidate driver of the stem-like CD8^+^ T cell program. Recent studies in cancer and chronic infection have identified IL-15 as a key regulator of stem-like progenitor–exhausted CD8^+^ T cells (TPEXs), promoting their self-renewal and long-term persistence (10). IL-15 signaling is among the expected targets of the JAK1/2 cytokine signaling inhibitor baricitinib, another promising immunotherapeutic agent for T1D (11). The therapeutic implications extend beyond JAK inhibition: mAbs targeting IL-15 itself (such as ordesekimab) have already entered clinical trials in autoimmune diseases (12), whereas mAbs blocking the shared IL-2/IL-15 receptor β chain (CD122) are under development, highlighting the translational potential of targeting this pathway (13) to disrupt the maintenance of stem-like autoreactive CD8^+^ T cells.

More broadly, the work by Peters et al. suggests that future therapies may need to target not only pathogenic effector T cells but also the PLN stem-like populations that replenish them. In this context, agonist PD-1 antibodies under clinical development (such as peresolimab; ref. 14) may shift the balance from stemness toward a clinically favorable exhaustion state, counter to the aim of cancer immunotherapies with checkpoint inhibitors.

## Knowledge gaps for future research

**Figure 1:**
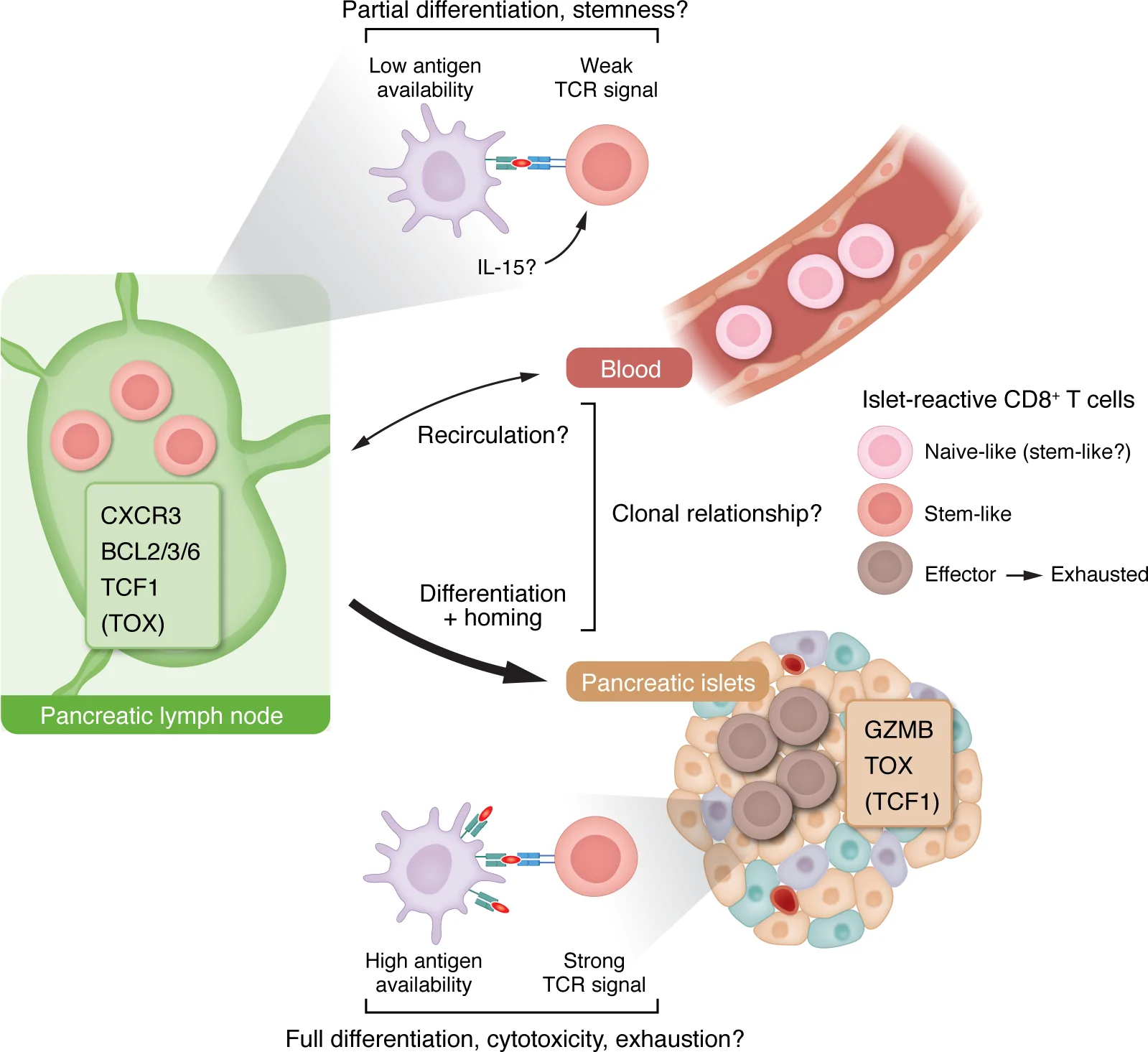
A proposed stemness-to-effector/exhaustion tissue axis in T1D. PLNs may serve as a reservoir for stem-like CD8^+^ T cells that sustain autoimmune responses in T1D. Building on the findings of Peters et al. (5) and previous observations in NOD mice, the model presented here proposes that limited β cell antigen availability in PLNs, combined with the intrinsically low affinity of autoreactive TCRs for β cell antigens, results in weak TCR signaling that favors partial differentiation and maintenance of stem-like properties. This state may be further supported by IL-15 signaling. Stem-like PLN CD8^+^ T cells (red) are characterized by the expression of CXCR3 and self-renewal–associated markers such as BCL2, BCL3, and BCL6, together with TCF1 and TOX. Whether circulating naive-like, islet-reactive CD8^+^ T cells (pink) represent a recirculating counterpart of this PLN cell population remains unknown. Upon migration to pancreatic islets, exposure to higher antigen loads and stronger TCR stimulation may promote further differentiation, acquisition of cytotoxic functions, and eventual exhaustion (brown cells). However, the developmental relationship between PLN stem-like cells and pancreatic effector/exhausted cells remains unresolved, as current evidence demonstrates phenotypic continuity but limited clonal overlap between the two compartments. The figure highlights key hypotheses and outstanding questions emerging from the study by Peters et al., including the roles of recirculation, clonal continuity, and the mechanisms governing the balance between stemness, effector differentiation, and exhaustion.

Several limitations of this work deserve consideration and provide inspiration for future research (Figure 1). First, as access to human PLN and pancreatic tissues remains rare, specimen numbers were modest, and so was the number of CD8^+^ T cells sampled, hampering a comprehensive dissection of T cell heterogeneity and antigen specificity. The inability to recover informative HLA class I dextramer signals highlights the challenge of tracking rare autoreactive T cells directly in human tissues. Although several TCR CDR3α sequences showed similarity to previously reported β cell–reactive clones, antigen specificity cannot be inferred with confidence. An intriguing aspect of the study is that the principal T1D-enriched, naive-like cell population was highly diverse and lacked evidence of clonal expansion. This observation contrasts with expectations derived from the NOD mouse model (3), in which stem-like autoreactive cells constituted an expanded reservoir of diabetogenic clones. Moreover, despite evidence of phenotypic continuity between PLN and pancreatic CD8^+^ T cell populations, direct clonal overlap between the two compartments was surprisingly limited. The absence of clonal expansion and the limited PLN-to-pancreas TCR sharing complicate direct extrapolation of the reservoir model proposed in NOD mice (3), where stem-like PLN CD8^+^ T cells were shown experimentally to sustain diabetogenic responses. Do the identified PLN populations represent the human counterpart of the murine diabetogenic reservoir or an earlier precursor state? Or do they reflect a broader T1D-associated remodeling of the CD8^+^ T cell compartment toward stemness? Future studies combining antigen-specific T cell tracking and paired multi-site TCR sequencing will be required to conclude whether these stem-like T cells constitute the developmental precursors of pathogenic islet-infiltrating effectors.

Second, the molecular signatures defining stemness remain variable across reports and disease settings. The relationships among stem-like TPEX and T stem cell memory (TSCM) states remain incompletely resolved, while the relative importance of canonical regulators such as TCF1, TOX, and other stemness-associated markers differs substantially across studies (15, 16). Furthermore, definitive demonstration that these stem-like T cells are nurtured by IL-15 signaling and directly generate pathogenic effectors in humans will require functional validation.

Third, is there a correlate of this stem-like phenotype in the blood that could provide a circulating T cell biomarker for T1D? Most studies have reported different degrees of exhaustion in circulating CD8^+^ T cells that correlate with disease activity (17, 18) and its therapeutic curbing (8, 19), i.e., the rates of progression from asymptomatic to clinical disease or of β cell loss after clinical onset. On the other hand, the description of different degrees of stemness is much more limited (20). B. Youngblood’s group has shown that circulating autoreactive T cells retain an epigenetically imprinted stem-like phenotype, intermediate between naive and effector/memory, that supports long-term persistence in T1D (21). Intriguingly, a predominantly naive-like (CD45RA^+^CCR7^+^) phenotype has also been described for both CD8^+^ T cells (1, 22) and CD4^+^ T cells (23) in blood, raising the possibility that this phenotype may hide intermediate stem-like states.

Fourth, what are the signals favoring the maintenance of stem-like properties? Autoreactive T cells feature low-affinity TCRs, which are likely exposed to low β cell antigen loads in PLNs (24) due to limited delivery in both soluble form (upon insulin granule exocytosis) (25, 26) and cell-bound form (ferried by antigen-presenting cells [APCs], in the frame of surface HLA molecules that also feature low binding affinity for β cell epitopes) (24). Overall, these features likely deliver very weak TCR signals, which may favor the maintenance of a partially differentiated stem-like phenotype in PLNs. This is also where the ancillary IL-15 signaling highlighted by Peters et al. may prove critical. Once partially differentiated T cells migrate to the pancreas, the higher antigen load available in the islet microenvironment may drive full differentiation into cytotoxic effector T cells and eventual exhaustion (24).

## Conclusions

Whether human PLNs truly harbor the self-renewing autoreactive reservoir postulated from murine studies remains an open question — one that will require more direct evidence of clonal and functional continuity between PLNs and the pancreas. Nonetheless, the study by Peters et al. supports the existence of a stemness-to-effector axis across human T1D tissues that expands our understanding of T1D pathophysiology and opportunities for therapeutic targeting.

## Conflict of interest

RM serves on advisory panels and speakers’ bureaus for Sanofi; financial compensation for these activities has been received by the AP-HP Foundation. RM receives research funding from Sanofi.

## Funding support

- The Leona M. and Harry B. Helmsley Charitable Trust (grant 2412-07951, to RM).
- Fondation pour la Recherche Médicale (FRM EQU202603051413, to RM).
- Agence Nationale de la Recherche (grant ANR-25-CE15-2811, to RM).
- European Foundation for the Study of Diabetes (EFSD) Young Investigator Award (to FS).

## Version 1 09/15/2026

Electronic publication

## References

[1] Culina S (2018). Islet-reactive CD8^+^ T cell frequencies in the pancreas, but not in blood, distinguish type 1 diabetic patients from healthy donors. Sci Immunol.

[2] Gagnerault MC (2002). Pancreatic lymph nodes are required for priming of beta cell reactive T cells in NOD mice. J Exp Med.

[3] Gearty SV (2022). An autoimmune stem-like CD8 T cell population drives type 1 diabetes. Nature.

[4] Im SJ (2016). Defining CD8^+^ T cells that provide the proliferative burst after PD-1 therapy. Nature.

[5] Peters LD (2026). Immune dysregulation and stem-like CD8^+^ T cell enrichment in type 1 diabetes pancreatic lymph nodes. J Clin Invest.

[6] Shapiro MR (2023). Human immune phenotyping reveals accelerated aging in type 1 diabetes. JCI Insight.

[7] Rachdi L (2023). Tryptophan metabolism promotes immune evasion in human pancreatic β cells. EBioMedicine.

[8] Long SA (2016). Partial exhaustion of CD8 T cells and clinical response to teplizumab in new-onset type 1 diabetes. Sci Immunol.

[9] Wu Y (2026). Anti-CD3 mAb treatment reshapes infiltrating T and β cells in the islets in autoimmune diabetes. JCI Insight.

[10] Lee J (2023). IL-15 promotes self-renewal of progenitor exhausted CD8 T cells during persistent antigenic stimulation. Front Immunol.

[11] Waibel M (2023). Baricitinib and β cell Function in Patients with New-Onset Type 1 Diabetes. N Engl J Med.

[12] Lähdeaho M-L (2019). Safety and efficacy of AMG 714 in adults with coeliac disease exposed to gluten challenge: a phase 2a, randomised, double-blind, placebo-controlled study. Lancet Gastroenterol Hepatol.

[13] Waldmann TA (2020). Interleukin-15 (dys)regulation of lymphoid homeostasis: Implications for therapy of autoimmunity and cancer. J Exp Med.

[14] Tuttle J (2023). A Phase 2 trial of peresolimab for adults with rheumatoid arthritis. N Engl J Med.

[15] Gebhardt T (2023). Stem-like exhausted and memory CD8^+^ T cells in cancer. Nat Rev Cancer.

[16] Beltra JC (2020). Developmental relationships of four exhausted CD8(+) T cell subsets reveals underlying transcriptional and epigenetic landscape control mechanisms. Immunity.

[17] McKinney EF (2015). T cell exhaustion, co-stimulation and clinical outcome in autoimmunity and infection. Nature.

[18] Wiedeman AE (2020). Autoreactive CD8+ T cell exhaustion distinguishes subjects with slow type 1 diabetes progression. J Clin Invest.

[19] Diggins KE (2021). Exhausted-like CD8^+^ T cell phenotypes linked to C-peptide preservation in alefacept-treated T1D subjects. JCI Insight.

[20] Vignali D (2018). Detection and characterization of CD8(+) autoreactive memory stem t cells in patients with type 1 diabetes. Diabetes.

[21] Abdelsamed HA (2020). Beta cell-specific CD8(+) T cells maintain stem cell memory-associated epigenetic programs during type 1 diabetes. Nat Immunol.

[22] Gonzalez-Duque S (2018). Conventional and neo-antigenic peptides presented by β cells are targeted by circulating naïve CD8^+^ T cells in type 1 diabetic and healthy donors. Cell Metab.

[23] Guyer P (2022). Recognition of mRNA splice variant and secretory granule epitopes by CD4^+^ T cells in type 1 diabetes. Diabetes.

[24] Samassa F, Mallone R (2022). Self-antigens, benign autoimmunity and type 1 diabetes: a beta cell and T cell perspective. Curr Opin Endocrinol Diabetes Obes.

[25] Wan X (2018). Pancreatic islets communicate with lymphoid tissues via exocytosis of insulin peptides. Nature.

[26] Azoury ME (2020). Peptides derived from insulin granule proteins are targeted by CD8^+^ T Cells Across MHC Class I restrictions in humans and NOD mice. Diabetes.

